# A DOX-loaded polymer micelle for effectively inhibiting cancer cells†

**DOI:** 10.1039/c8ra04089c

**Published:** 2018-07-19

**Authors:** Huayang Feng, Dandan Chu, Zhanrong Li, Zhihua Guo, Lin Jin, Bingbing Fan, Junjie Zhang, Jingguo Li

**Affiliations:** People's Hospital of Zhengzhou University, Zhengzhou University Henan 450003 China lijingguo@zzu.edu.cn +86-371-65580918; School of Material Science and Engineering, Zhengzhou University Henan 450001 China fanbingbing@zzu.edu.cn +86-371-67782176

## Abstract

A novel triblock polymer is synthesized and self-assembled with doxorubicin to form DOX-loaded micelles. The synthetic process involves the ring-opening polymerization, carboxylation and amidation reactions, and the structures are characterized. The drug release test indicated that the micelles have the ability to control the release of drugs. The cell uptake results indicated that the DOX-loaded micelles could enter cancer cells easily, and the cytotoxicity and apoptosis test confirmed that DOX-loaded micelles have a strong killing effect on tumor cells, while the blank micelles do not have cytotoxicity. Therefore, the novel polymer micelles are a promising carrier for delivery of anticancer drugs to enhance cancer treatment.

## Abstract 2

A novel triblock polymer is synthesized and self-assembled with doxorubicin to form DOX-loaded micelles.

## Graphical abstract

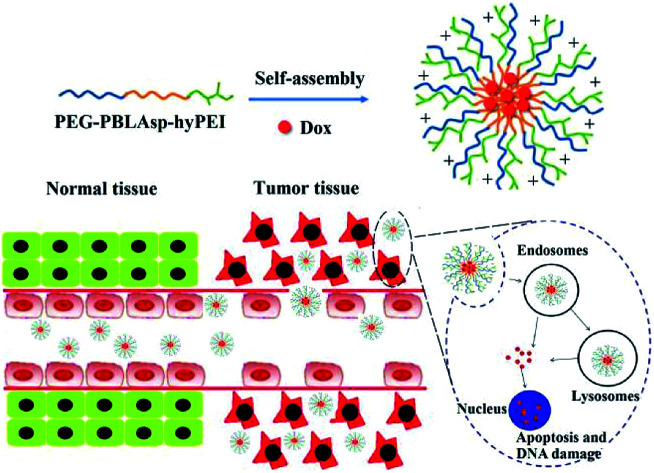

## 1. Introduction

Malignant tumors have become one of the most important threats to human health in recent years.^1,2^ The main methods employed to treat cancer in the clinic are chemotherapy, radiotherapy and surgery. Among them, surgery and radiation therapies can only treat localized cancer, while chemotherapy is able to treat widespread cancer.^3^ Doxorubicin (DOX), also known as adriamycin (ADR), is a potent chemotherapeutic drug applied in the clinic for the treatment of a wide range of human cancers, including Hodgkin's lymphoma, leukemia, multiple myeloma, breast cancer, osteosarcoma, ovarian cancer and lung cancer. Like other anthracyclines, DOX takes effect by intercalating DNA in cancer cells and inhibiting macromolecular biosynthesis.^4^ However, under neutral and alkaline conditions, doxorubicin is hydrophobic, and only under acidic conditions, doxorubicin can be converted to hydrophilic and partially dissolved into water. So the poor water solubility at neutral and alkaline conditions and the great toxic side effects are the key factors hindering its clinical application.

In this regard, researchers have developed a variety of drug delivery systems (mesoporous silica,^5^ gold nanoparticles,^6^ and polymer micelles,^7–9^*etc.*) to improve the poor water solubility and high toxicity of doxorubicin and other chemotherapy drugs. Among various nanocarriers for drug delivery, polymer micelles assembled from amphiphilic block copolymers have attracted widespread attention due to their adjustable size and ability to increase the water solubility of hydrophobic drugs.^10,11^ In addition, polymer micelles can also enrich the chemotherapeutic drugs in tumor sites through a mechanism known as the enhanced permeability and retention (EPR) effect.^12–14^

However, most chemotherapeutic drugs do not exert anti-tumor effects outside the cell, so it is crucial to develop polymeric micelles that promote endocytosis of chemotherapeutic drugs. In this regard, researchers have designed active targeting of nanomedicine to enhance endocytosis of cells. The most common strategy is to attach some targeting ligands (folate,^15,16^ antibody,^17^ RGD,^18^*etc.*) to the surface of polymeric micelles. Targeted ligands, although increasing endocytosis of tumor cells, reduce micellar stability and some ligands are expensive and difficult to synthesize, so we designed a triblock cationic drug-loaded micelle to enhance cellular endocytosis. The polymer is composed of a hydrophilic polyethylene glycol (PEG) block and a hydrophobic poly(β-benzyl l-aspartate) (PBLAsp) and a branched poly(ethylenimine) (PEI) block.

Nowadays, micelles based on poly(ethylene glycol) (PEG) have drawn great attention in cancer therapy due to their distinct advantages in delivery of anticancer drugs which can improve the solubility and stability of hydrophobic antitumor drugs and prolong *in vivo* circulation time.^19,20^ As we all know, polyethyleneimine (PEI) has a proton sponge effect which could cause the lysosomal swelling to rupture, and then release drugs into the cytoplasm, further leading to mitochondrial damage and apoptosis.^21,22^ More importantly, a polymer micelle must be biodegradable, similar to the structure of natural proteins, PBLAsp have a good biodegradability and biocompatibility and is widely used in drug controlled release carrier research.^23,24^ So we designed a triblock DOX-loaded micelle for cancer therapy. The polymer is composed of a hydrophilic PEG block and a hydrophobic PBLAsp block and a branched PEI block.

**Fig. 1:**
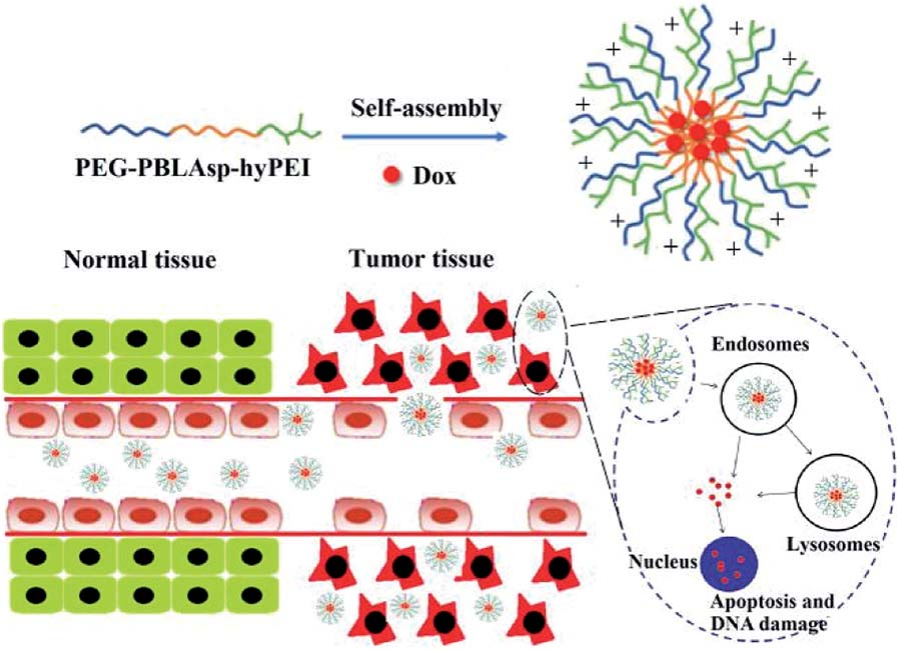
Illustrative preparation of micelle as well as the release of DOX inside tumor cell.

Herein, a triblock polymer PEG-PBLAsp-hyPEI (PEAhI) is synthesized by ring-opening polymerization, carboxylation and amidation reactions and self-assembled with doxorubicin to form triblock drug-loaded micelles (Fig. 1). As a hydrophilic shell, PEG provides a good stealth for micelles and hyPEI makes the micelles positively charged and helps the micelles enter the tumor cells. PBLAsp as a hydrophobic core could effectively support doxorubicin and make the micelle biodegradable. The physicochemical properties and drug release performance of the DOX-loaded micelle were investigated. The anticancer activity of DOX-loaded micelle was examined in human hepatocarcinoma SMMC-7721 cells and human breast adenocarcinoma MCF-7 cells.

## 2. Materials and methods

### 2.1 Materials

*N*-Carboxyanhydride of β-benzyl-l-aspartate (BLAsp-NCA) was synthesized as previously reported.^20^ The following reagents were of analytical grade and used as received: α-methoxy-ε-amino poly(ethylene glycol) (mPEG-NH_2_, *M*_n_ = 2 kDa), succinic anhydride (SA, Sigma-Aldrich), dicyclohexylcarbodiimide (DCC, Sigma-Aldrich), *N*-hydroxysuccinimide (NHS, Sigma-Aldrich) and branched polyethyleneimine (hyPEI, *M*_n_ = 1.8 kDa). Dialysis bag (*M*_w_ cut-off: 3.5 kDa) was purchased from Shanghai Green Bird Technology Development Co., Ltd. China. Chloroform (CHCl_3_), ethyl acetate (C_4_H_8_O_2_) and petroleum ether were dried over CaH_2_ and then distilled under ambient pressure. All other reagents were of analytical grade and used as received. SMMC-7721 and MCF-7 cells were obtained from the Kunming cell library of the Chinese Academy of Sciences. Cell counting Kit-8 (CCK-8) was purchased from Beyotime. TUNEL apoptosis test kit was purchased from KeyGEN bioTECH.

### 2.2 Synthesis of polymers (PEAhI)

#### 2.2.1 Synthesis of mPEG-PBLAsp

Poly(ethylene glycol)-*block*-poly(β-benzyl-l-aspartate) was synthesized by ring-opening polymerization of *N*-carboxy anhydride of β-benzyl-l-aspartate (BLAsp-NCA) using mPEG-NH_2_ as a macroinitiator as reported.^21^ Briefly, PEG-NH_2_ (2.0 g, 1 mmol) was vacuum-dried in a 50 mL flask at 70 °C for 4 h before 20 mL of anhydrous DMF was added to dissolve mPEG-NH_2_. After BLAsp-NCA (9.9 g, 39.8 mmol) was dissolved in 10 mL of anhydrous DMF and then added into the above solution under the protection of nitrogen, the reaction was allowed to proceed for 72 h at 35 °C. Subsequently, the reaction mixture was precipitated into a large amount of cool diethyl ether, filtered, washed with diethyl ether, and dried under vacuum for 24 h until a constant weight was attained. (*M*_n_ = 10.2 kDa, calculated from ^1^H NMR spectrum, mPEG-PBLAsp).

#### 2.2.2 Synthesis of mPEG-PBLAsp-COOH

mPEG-PBLAsp-NH_2_ (5.0 g, 0.5 mmol) was dissolved in 50 mL of anhydrous chloroform, and then succinic anhydride (0.6 g, 6.0 mmol) was added into the reaction system under a nitrogen atmosphere. The reaction was allowed to proceed at 70 °C for 72 h. After adding 5 mL of ultrapure water, the reaction was continued for another 30 minutes. Subsequently, the reaction mixture was precipitated 2 times into a large amount of ethanol, filtered, washed with anhydrous ethanol, and finally dried under vacuum for 24 h until a constant weight was attained. (*M*_n_ = 10.3 kDa, calculated from ^1^H NMR spectrum, mPEG-PBLAsp-COOH).

#### 2.2.3 Synthesis of mPEG-PBLAsp-hyPEI

mPEG-PBLAsp-COOH (1.0 g, 0.1 mmol) and NHS (0.014 g, 0.12 mmol) were dissolved in 5 mL of anhydrous DMSO and reacted for 2 h at room temperature. DCC (0.023 g, 0.12 mmol) and hyPEI (0.75 g, 0.3 mmol) were dissolved in 10 mL of anhydrous DMSO and then slowly added into the above solution. The reaction was performed for 24 h at room temperature. The precipitated 1,3-dicyclohexylurea (DCU) was removed by filtration, and the filtrate was dialyzed against water using dialysis bag (*M*_w_ cut-off: 3.5 kDa) for 3 days and then freeze-dried by a lyophilizer to obtain mPEG-PBLAsp-hyPEI (*M*_n_ = 12 kDa, calculated from ^1^H NMR spectrum).

### 2.3 Preparation of micelle

30.0 mg of mPEG-PBLAsp-hyPEI and 3.0 mg DOX were dissolved in 5 mL chloroform, and then the solution was added dropwise to PBS at pH 7.4 (30 mL) under ultrasonic homogenizer using a Type CPX 600 (Cole-Parmer, America) at a power level of 40%. After chloroform was removed by rotary evaporation, the solution was adjusted to pH 7.4. Afterwards, the solution was filtered with a syringe filter (pore size: 0.45 μm) to eliminate aggregates, concentrated, and washed three times using a MILLIPORE Centrifugal Filter Device (*M*_w_ cut-off: 30 000 Da)

### 2.4 Characterization of the polymer and micelle

^1^H NMR spectra were obtained using a Bruker AVANCElll 400 MHz spectrometer. DMSO-d_6_ was used as the solvent depending on polymer solubility.

FTIR spectral studies were carried out using an IS10 670 FTIR spectrometer in the range between 4000 and 500 cm^−1^ at a resolution of 2 cm^−1^. All powder samples were compressed into KBr pellets in the FTIR measurements.

Atomic Force Microscope (AFM) was performed using a Bruker Nanoscope VIII Multi-Mode with a “J” scanner (scan range: 125 μm × 125 μm × 5 μm) and operated in PeakForce Tapping Mode using Bruker silicon nitride probes (Model: SCANASYST-AIR, *f*_o_ = 150 kHz, *k* = 0.4 N m^−1^, length = 115 μm, width = 25 μm, tip radius: ∼2 nm) at room temperature in air conditioning. The samples were prepared by drying a drop (10 μL, 1 mg mL^−1^) of the sample solution on a mica slice. The mica slice was finally dried overnight in a desiccator before AFM observation. All the images were “flatten” by the AFM software Nanoscope Analysis V1.40 without other processing.

The hydrodynamic sizes were determined *via* dynamic light scattering (DLS). Measurements were performed at 25 °C using a Nano-ZS90 equipment (Malvern Instruments Corporation, UK). The data was collected on an auto-correlator with a detection angle of scattered light at 90°. For each sample, the data from three measurements were averaged to obtain the mean ± standard deviation (SD).

### 2.5 *In vitro* DOX release from micelle

Micelle solutions at a certain concentration in PBS at pH 7.4 and pH 5.0 were transferred into a dialysis bag (*M*_w_ cut-off: 3500 Da). The bag was placed into the same buffered solution (150 mL), and the release study was performed at 37 °C in an incubator shaker (TS-100C, Shanghai Kuangbei, China). At certain time intervals, 3 mL of solution outside the dialysis bag was replaced with the same volume of fresh buffer solution for UV-vis analysis. DOX concentration was calculated based on the absorbance intensity of DOX at 497 nm. In the assessment of drug release, the cumulative amount of released drug was calculated, and the percentages of drug released from micelles were plotted against time.

### 2.6 Confocal laser scanning microscopy (CLSM)

The cellular uptake of DOX-loaded micelle in SMMC-7721 and MCF-7 cells was observed on a single-photon confocal laser scanning microscope (Nikon C1si, Japanese). SMMC-7721 or MCF-7 cells were seeded in 20 mm glass bottom Petri dishes at a density of 1 × 10^3^ cells per dish and incubated overnight at 37 °C in 1 mL of RPMI1640 or MEM medium containing 10% fetal bovine serum (FBS) and then incubated with micelles (DOX concentration: 0.4 μg mL^−1^) for different time (1, 2, 4, 6 h). The cells were washed twice with PBS, and then stained with Hoechst 33342 solution (10 μg mL^−1^) for 15 min for CLSM observation. The excitation and emission wavelengths of DOX and Hoechst are 488 nm and 590 nm, 350 nm and 460 nm, respectively.

### 2.7 Cytotoxicity evaluated by CCK-8 assay

100 μL of MCF-7 or SMMC-7721 cell suspension was prepared in 96-well plates. The plates were pre-incubated in an incubator for 24 hours (at 37 °C under 5% CO_2_). The cells were incubated with DOX-loaded micelles and blank micelles at various concentrations for 48 h before being measured with CCK-8 assay. After replacing the medium in each well with 100 μL fresh medium containing 10 μL CCK-8 solutions, the cells were incubated for additional 4 h at 37 °C. The absorbance at 450 nm was then detected using an Enzyme Labeler (PerkinElmer EnVision, England). The cell viabilities were calculated by comparing absorbance with negative control. All experiments were conducted in triplicate.

### 2.8 Cell apoptosis by TUNEL

The cell apoptosis rates in different treatment groups were also detected with TUNEL assay. MCF-7 or SMMC-7721 cells were seeded into 6-well plates at a density of 2 × 10^3^ cells per well and cultured overnight. And the cells were incubated with different treatment samples for 24 h, 48 h and 72 h. The KGA TUNEL Apoptosis Detection Kit (KeyGEN bioTECH, China) was used to estimate the percentage of apoptotic cells according to the manufacturer's protocol. In brief, cells were fixed with 200 μL of 4% paraformaldehyde for 20 min at room temperature and then washed three times with PBS. Then the cells were promoted seepage 3–5 min using 1% Triton X-100 permeabilized liquid and washed three times with PBS. Afterwards, the cells were blocked with 3% H_2_O_2_ blocking solution for 10 min at room temperature and washed three times with PBS. Then each sample was added 50 μL of POD-conjugated anti-FITC working solution and placed in a warm box at 37 °C for 30 min in the dark. After washed with PBS three times, each sample was added 50 μL DAB liquid at room temperature. After chromogenic reaction for 30 s to 5 min and washed, the samples were washed with PBS three times and stained with hematoxylin for 30 s to 5 min. The samples were rinsed with distilled water and then immersed in 1% hydrochloric acid in methanol for 5 s and rinsed with distilled water immediately. At last, the samples were immersed in ethanol for 5 min and dipped with xylene twice for 10 min each time. After being dried, the samples were added neutral gum and covered with a glass slide before being observed using a fluorescence microscopy (Nikon ECLIPSE 80i, Japanese).

## 3. Results and discussion

### 3.1 Polymer synthesis and characterization

**Fig. 2:**
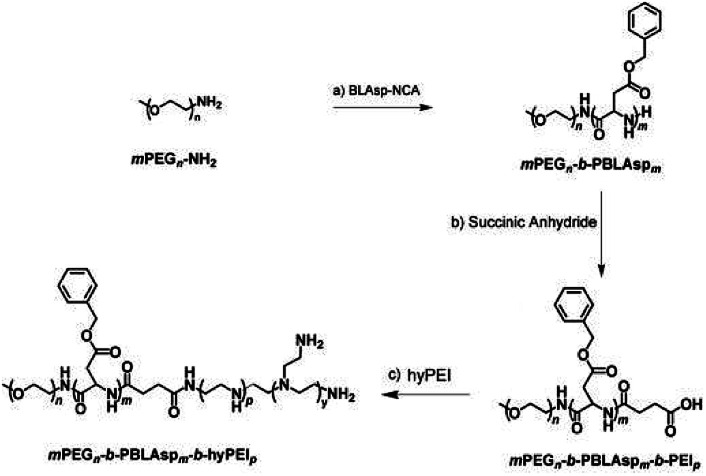
Synthesis steps of the triblock polymer PEG-PBLAsp-hyPEI.

**Fig. 3:**
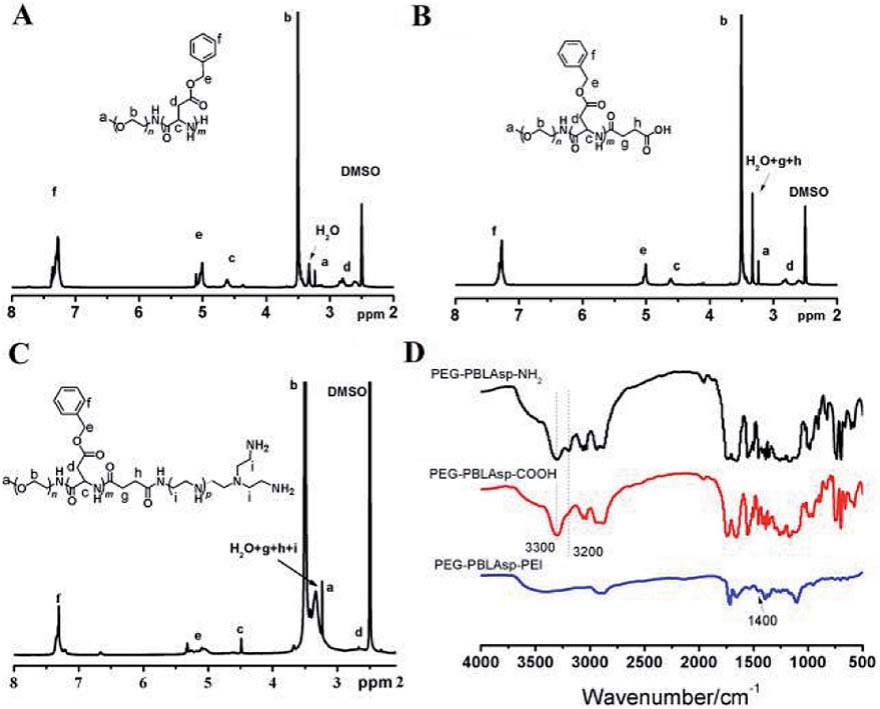
^1^H NMR spectra of PEG-PBLAsp-NH_2_ (A), PEG-PBLAsp-COOH (B) and PEG-PBLAsp-hyPEI (C) in DMSO. FTIR spectra (D) of PEG-PBLAsp-NH_2_, PEG-PBLAsp-COOH and PEG-PBLAsp-hyPEI.

The synthetic approach of PEG-PBLAsp-hyPEI is illustrated in Fig. 2. And the chemical structure of polymer was verified by ^1^H NMR and FTIR analyses (Fig. 3). The degrees of polymerization for the PBLAsp blocks were 40.

Fig. 3A shows the ^1^H NMR spectrum of mPEG-PBLAsp in DMSO-d_6_, and the assignment of the resonances in the ^1^H NMR spectrum of that showed characteristic peaks at 2.82 ppm (–C**H**_**2**_COO–), 3.33 ppm (–OC**H**_**3**_), 3.62 ppm (–OC**H**_**2**_C**H**_**2**_–), 4.62 ppm (–COC**H**NH–), 5.01 ppm (–COOC**H**_**2**_C_6_H_5_), 7.25 ppm (–COOCH_2_C_6_**H**_**5**_), indicating the successful synthesis of the target product. The average degree of polymerization (DP) of the PBLAsp segment was 40 by calculating the peak intensity ratio of the CH_2_ group of PEG at 3.62 ppm and the C_6_H_5_ group of at PBLAsp 7.25 ppm.

After the carboxylation reaction, in addition to maintaining the characteristic peaks of the precursor polymer, the characteristic peaks of the methylene groups above the succinic acid are added (Fig. 3B). In the FTIR spectrum, the disappearance of a characteristic peak of primary ammonia around 3200 cm^−1^ further confirmed the formation of PEG-PBLAsp-COOH (Fig. 3D).

The ^1^H NMR spectrum of the final product is shown in Fig. 3C. In addition to maintaining the characteristic peak of the PEG-PBLAsp-COOH precursor block copolymer, a broad PEI peak appeared at 3.0–3.4 ppm, indicating the successful synthesis of the target product. In the FTIR spectrum, the new characteristic peaks appearing around 1400 cm^−1^ are assigned to PEI, which further confirmed the formation of the final product.

### 3.2 Preparation and characterization of micelle

**Fig. 4:**
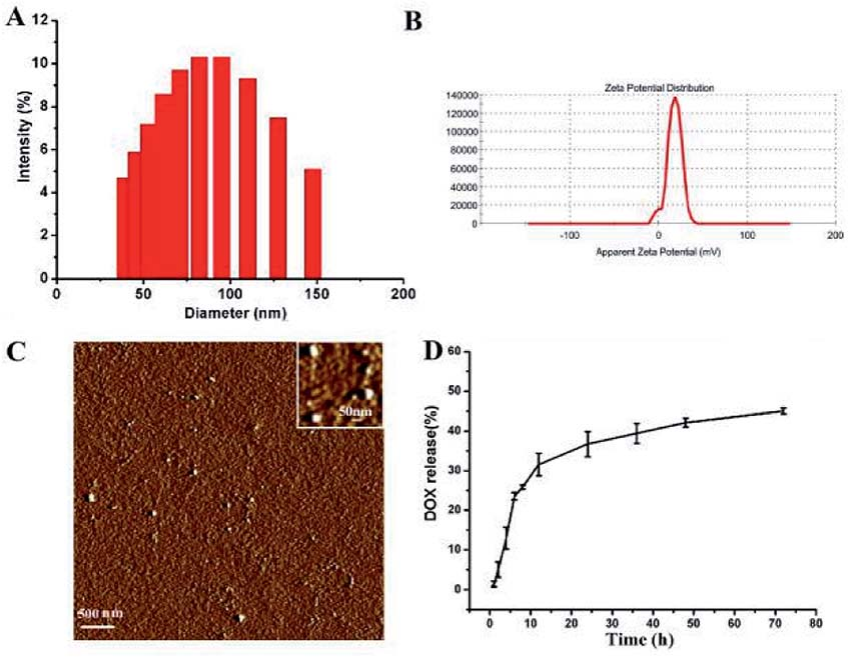
The size distribution (A) and zeta potential (B) and AFM image (C) of the blank micelles. *In vitro* DOX release profiles of DOX-loaded micelle at 37 °C in PBS solutions at pH 7.4 (D).

The size and zeta-potential of these micelles were evaluated using DLS. As shown in Fig. 4, the blank micelles exhibited an averaged hydrodynamic size of 80 nm and a zeta potential of +17.9 mV. Atomic Force Microscope (AFM) was employed to further visualize the formation and morphology of the micelles.

The DOX-loading content in the polymer micelles was 5.5%, which was calculated using previously established calibration curves. The size and size distribution of the blank and DOX-loaded micelles were analyzed by DLS. And the mean diameter of the DOX-loaded micelle was 81 nm (ESI Fig. 2†). The DLS results indicated that the particle size did not change substantially after the DOX was loaded.

### 3.3 *In vitro* release of DOX

The drug release of polymer micelles was evaluated by measuring the absorbance intensity of DOX at 497 nm of solutions. And the release profiles of DOX from micelles were conducted at different pH conditions *in vitro*. As shown in Fig. 4D, at pH 7.4, the release rate of DOX was very slow, nearly 30 wt% of the encapsulated DOX was released at 12 h, and more than 40 wt% of the DOX was released in 48 h. When the pH value of micelle solution was changed to 5.0, the release rate of DOX was a little faster than that of pH 7.4 (ESI Fig. 1†). This DOX release behavior indicates that our micelles have the ability to control the release of drugs.

### 3.4 Cellular uptake and intracellular distribution of micelle

**Fig. 5:**
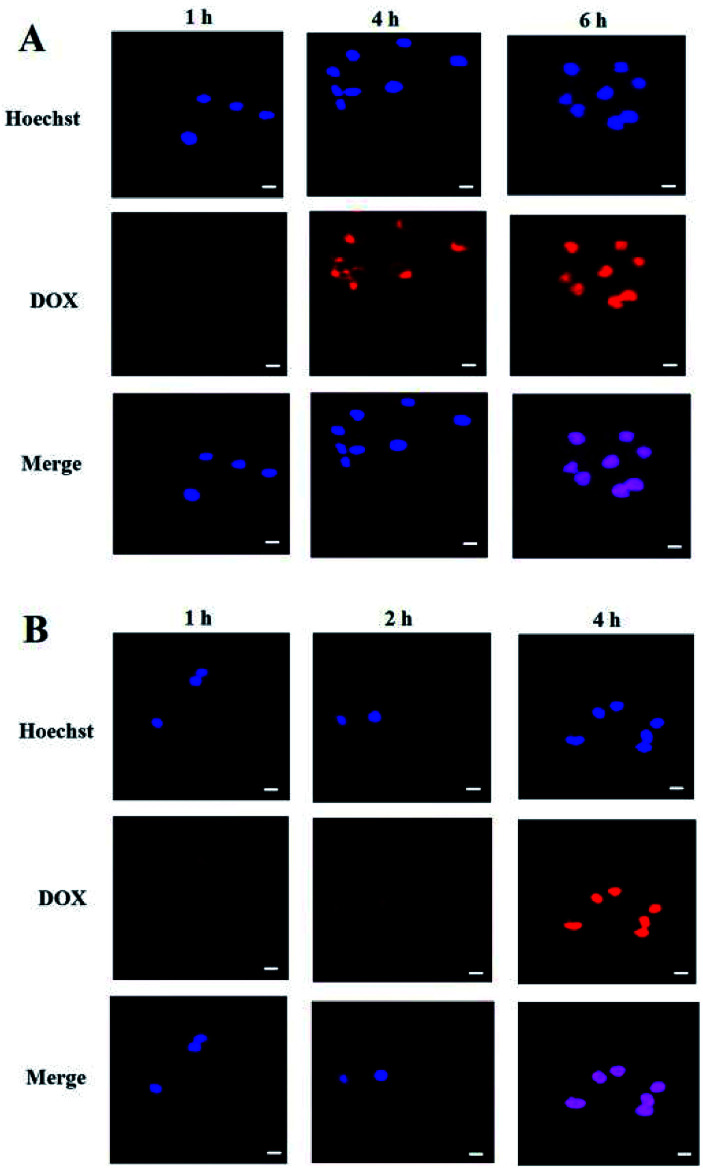
Confocal laser microscopic images of (A) SMMC-7721 cell and (B) MCF-7 cell incubated with DOX-loaded micelle at different time points. Red fluorescence: DOX; blue fluorescence: nuclei stained with Hoechst. The bar was 10 μm.

Cell uptake and intracellular DOX release of the PEG-PBLAsp-hyPEI micelles were evaluated with confocal laser scanning microscopy (CLSM) (Fig. 5). The results were obtained in SMMC-7721 and MCF-7 cells. Effective cell uptake of micelle was observed in both cells. Moreover, red DOX fluorescence was observed in the cell nuclei just after 2 h cell incubation with the micelles. As the cell incubation time extended, more DOX migrated to the nuclei from cytoplasm. After 4 h cell incubation, strong red fluorescence of DOX was nearly only observable in nuclei of MCF-7 cells. And after 6 h did the drug also fully enter SMMC-7721 cells' nucleus. The time when the drug completely enters the cells' nucleus is two hours earlier than previously reported.^19,24^ These results indicated that DOX-loaded micelle could quickly enter the cells and DOX was quickly released from the micelles inside the cell, and then migrated into the nuclei.

### 3.5 Cytotoxicity

**Fig. 6:**
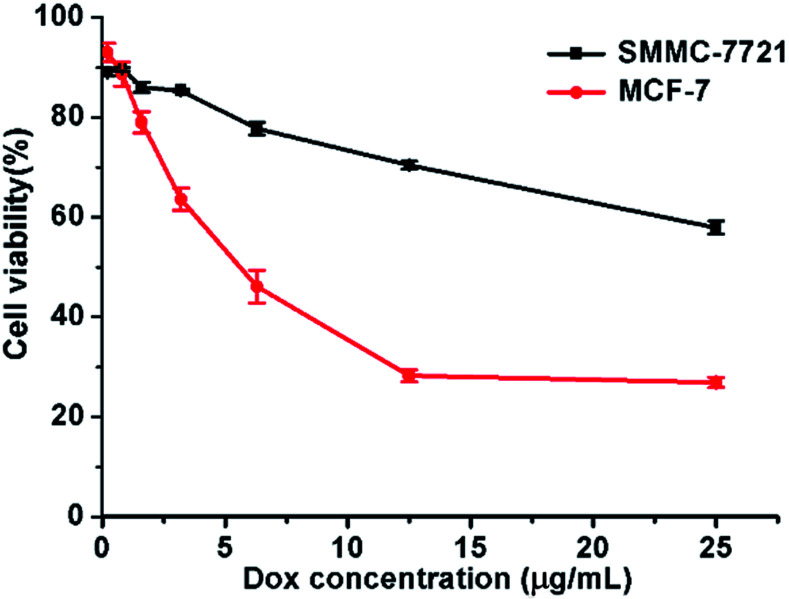
Cytotoxicity of SMMC-7721 and MCF-7 cells incubated with DOX-loaded micelles. Data were detected by CCK-8 assay. Incubation time: 48 h.

The cytotoxicity of micelle was determined by CCK-8 assay in SMMC-7721 and MCF-7 cells. Cells were treated for 48 h at various DOX concentrations ranging from 0.2 to 25 μg mL^−1^. As shown in Fig. 6, for both cells, the cell viability decreased obviously as the micelle concentration increased. At the highest investigated DOX concentration (25 μg mL^−1^), the viabilities of SMMC-7721 and MCF-7 cells further dropped to just 57.9 ± 1.3% and 26.9 ± 1.1%, respectively. The IC_50_ for MCF-7 cells was approximately 6.3 μg mL^−1^. We estimate the IC_50_ of DOX-loaded micelles for SMMC-7721 cells as approximately 40 μg mL^−1^ according to the trend of the curve. And the IC_50_ for both breast cancer and liver cancer cells was much lower than that of “free” DOX, which is approximately 40 μg mL^−1^ for breast cancer cells and 1 mg mL^−1^ for liver cancer cells. And the IC_50_ for both breast cancer and liver cancer cells was also much lower than part of other studies.^25,26^ And blank micelles showed lower cytotoxicity at this concentration because the PEG block overcomes the cytotoxicity of hyPEI^27^ (ESI Fig. 3†). These results are consistent with the cell uptake and intracellular DOX release data. These results showed the application potential of DOX-loaded micelles in cancer therapy.

### 3.6 Cell apoptosis

**Fig. 7:**
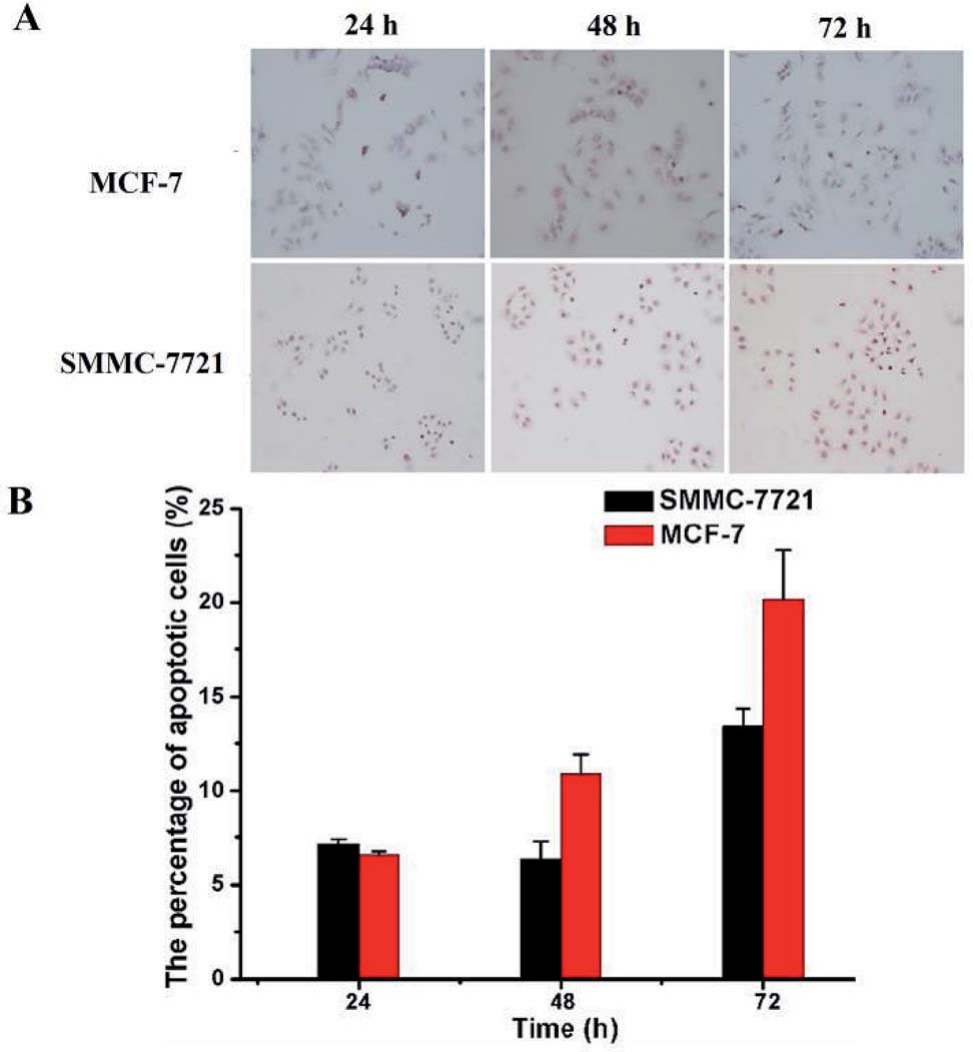
The apoptosis of MCF-7 and SMMC-7721 cells was evaluated by TUNEL assay. MCF-7 cells were incubated with DOX-loaded micelles for 24 h, 48 h, 72 h, SMMC-7721 cells were incubated with DOX-loaded micelles for 24 h, 48 h, 72 h (A). The nuclei of apoptotic cells were stained brown. The statistical apoptosis rate was shown in (B).

Finally, TUNEL assay was conducted to reveal whether the above mentioned DOX-loaded micelles on SMMC-7721 and MCF-7 cells may result in enhancement of cell apoptosis. As shown in Fig. 7, the nuclei of the apoptotic cells were stained brown, and with the prolongation of the drug's action time (D0X 0.8 μg mL^−1^), the rate of apoptosis of two kinds of cells showed an increasing trend. And under the same drug action time (72 h), MCF-7 cells have a higher apoptosis rate than that of SMMC-7721 cells (19.68% *vs.* 13.43%), which was consistent with the CCK-8 results.

## 4. Conclusions

In conclusion, a novel polymer, PEG-PBLAsp-hyPEI, was successfully synthesized by ring-opening polymerization, carboxylation and amidation reactions, and the successful synthesis was confirmed by ^1^H NMR and FT-IR. DLS measurement showed that the copolymer could self-assemble into micelles with a diameter of 80 nm in aqueous solution and AFM also confirmed the results. Drug release experiments proved that the DOX-loaded micelles have the ability to control the release of drugs. *In vitro* studies show that the DOX-loaded micelles may effectively enter cancer cells and then quickly release DOX to exert anticancer activity. These features make the DOX-loaded PEG-PBLAsp-hyPEI micelles promising in cancer therapy.

## Conflicts of interest

There are no conflicts to declare.

## Supplementary Material

RA-008-C8RA04089C-s001
