# Supplementary material for: A DOX-loaded polymer micelle for effectively inhibiting cancer cells
Source: RSC Adv. 2018 Jul 19;8(46):25949–54. doi: 10.1039/c8ra04089c (PMC9082780; doi:10.1039/c8ra04089c)
Supplement: RA-008-C8RA04089C-s001 [file RA-008-C8RA04089C-s001.pdf]

Electronic Supplementary Material (ESI) for RSC Advances.
This journal is © The Royal Society of Chemistry 2018

A DOX-loaded polymer micelle for effectively inhibiting cancer cells

Huayang Feng,[ab] Dandan Chu,[a] Zhanrong Li,[a] Zhihua Guo,[a] Lin Jin,[a] Bingbing Fan,*[b] Junjie Zhang[a] and Jingguo Li*[ab]

a People's Hospital of Zhengzhou University, Zhengzhou University, Henan 450003, China.

b School of Material Science and Engineering, Zhengzhou University, Henan 450001, China.

**** Corresponding Author:***

Bingbing Fan: School of Material Science and Engineering, Zhengzhou University, Henan 450001, China. E-mail: fanbingbing@zzu.edu.cn, Tel: +86-371-67782176.

Jingguo Li: Zhengzhou University People's Hospital, School of Material Science and Engineering, Zhengzhou University, Henan 450001, China. E-mail: lijingguo@zzu.edu.cn; Tel: +86-371-65580918.

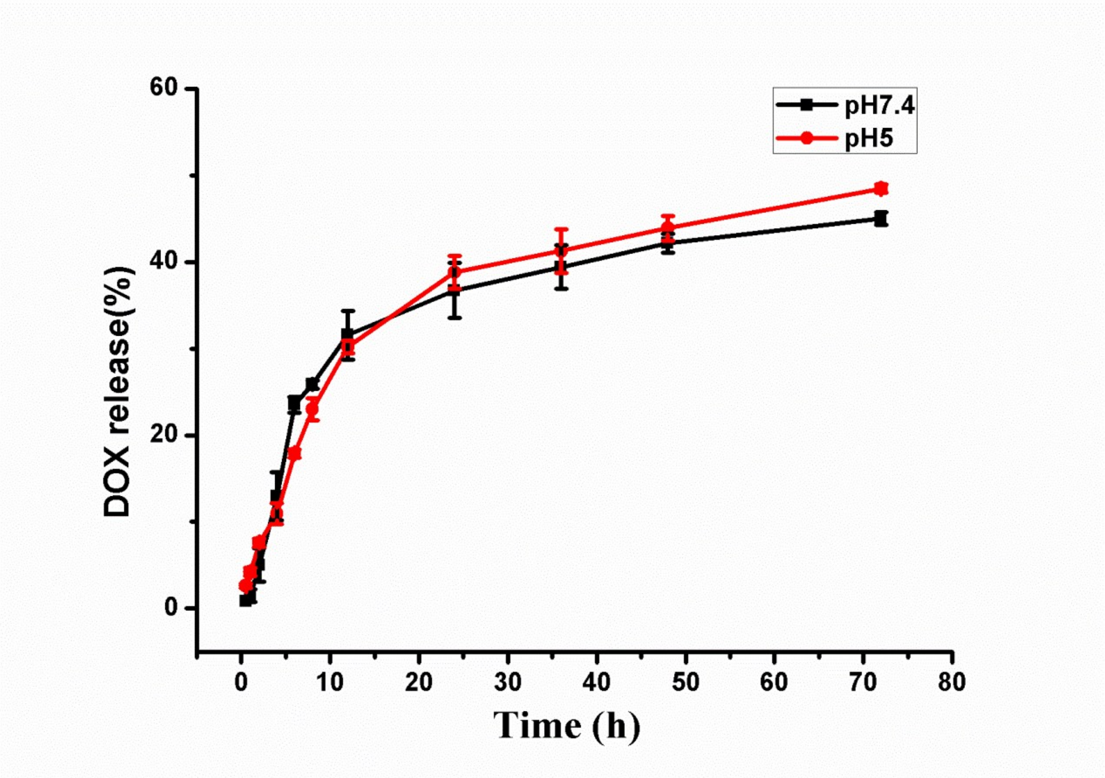

ESI Fig. 1† in vitro DOX release profiles of drug-loaded micelle at 37 °C in PBS solutions at pH 7.4 and 5.0

We did this experiment for three times, and found little difference between pH7.4 and pH5.0. It may be explained by the following two reasons. First, there is no acid-sensitive group in this polymer, so the drug release is insensitive to the changes in pH. Second, because the sensitivity of the measurement equipment, measurement errors may occur, and these errors are greater than the range of drug release changes.

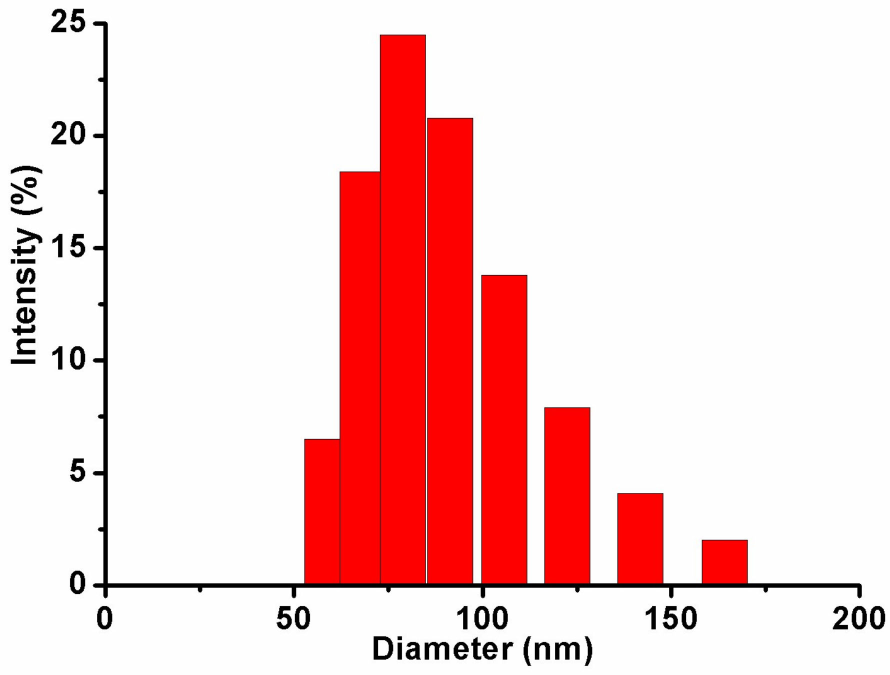

ESI Fig. 2† The size distribution of the DOX-loaded micelles

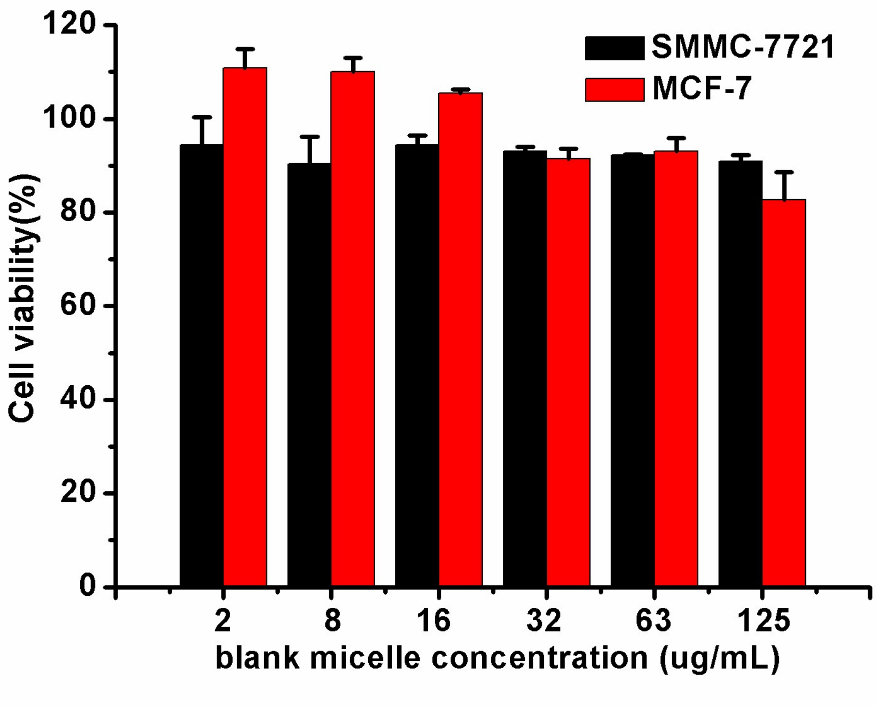

ESI Fig. 3† Cytotoxicity of SMMC-7721 and MCF-7 cells incubated with blank micelle. Data were detected by CCK8 assay. Incubation time: 48 h
